# Community as the key to socio-education: an analysis of challenges and practices in migratory contexts

**DOI:** 10.3389/fsoc.2025.1646111

**Published:** 2026-01-05

**Authors:** Massimo Santoro, Almudena Iniesta Martínez, Práxedes Muñoz Sánchez, Daniele Battista, Domenico Santaniello

**Affiliations:** 1 Catholic University of Murcia, Murcia, Spain; 2 University of Salerno, Fisciano, Italy

**Keywords:** reception center, socio-educational agents, unaccompanied foreign minors, social work, educational tools

## Abstract

**Introduction:**

This study examines socio-educational work in reception centres for unaccompanied foreign minors (MENAS) in Salerno, Italy, focusing on tools, practices, and challenges within residential care.

**Methods:**

Qualitative single-case study; interviews with judges, prosecutors, law enforcement and staff; two focus groups with eight socio-educational workers; thematic analysis using a grounded approach.

**Results:**

Participants reported tensions between empathy and professional distance, bureaucratic hurdles, language barriers, trauma-related needs, and limited inter-institutional coordination. Six thematic categories emerged: actors’ map; migration drivers; challenges; practitioner role; minors’ perspectives; operational tools.

**Discussion:**

Findings align with social learning, scaffolding, and transference frameworks. Reflective teamwork and structured daily activities support integration but require supervision and training. Implications concern inter-agency coordination and rights-based inclusion pathways.

## 1 Introduction

The present study has been conducted to examine the lives and work of socio-educational agents in centers for unaccompanied foreign minors. The objective of the study is twofold: firstly, to understand the support tools available to agents, and secondly, to reflect on residential intervention. The study focuses on the case of minors in Salerno, southern Italy, analyzing social, cultural, political, and economic influences (Yin, 2014). The exploratory approach is based on the case study, allowing for the answering of the “how” and “why” of specific phenomena (Edmondson and McManus, 2007). The research encompassed the “Neverland” reception center of the *Giovamente* social cooperative, which is authorized by Law 184/1983 and amendments of Law 149/2001. Judges, prosecutors, and law enforcement agencies were interviewed to address legal aspects and collect statistical data on minors. Community challenges, characterized by complex relationships and limited connections, were also explored (Cottrell, 2013). The children originate from contexts of poverty and conflict, seeking a better future. Socio-educational actors face challenges and must maintain appropriate distances to foster effective interactions (Banks, 2012). They are role models where active listening (Bandura, 1977), empathy, and cultural knowledge are essential for educational success (Neri, 2019). The present study does not aim to generalize but rather to understand the uniqueness and complexity of the context, thereby promoting reflections on social and educational intervention (Stake, 2005). Italy’s strategic geographical position has rendered it a primary destination for migrants, including unaccompanied foreign minors (MENAS). The migratory journey is intricate, entailing the disconnection from familial, cultural, and historical affiliations. This involuntary separation engenders profound vulnerability, which is exacerbated upon arrival in the host country, particularly during the most sensitive and formative phases of adjustment [United Nations High Commissioner for Refugees (UNHCR), 2022; International Organization for Migration (IOM), 2020]. For a considerable number of individuals, the decision to leave their country of origin is driven by the need to escape conflict, poverty, violence, or abuse. Europe is perceived as a potential site for the reconstruction of lives, the identification of opportunities, and the evasion of inhumane conditions. The response to this phenomenon necessitates a comprehensive approach addressing the material and socio-emotional dimensions involved. It is observed by United Nations Children’s Fund (UNICEF) (2024) that there was a considerable increase in migratory flows to Italy in the early 2000s, particularly following the dissolution of short-term visits, which resulted in many migrants overstaying. The Italian reception system has undergone persistent alterations in its regulations, procedures, and reception structures, constantly evolving to adapt to novel challenges and emerging situations. In recent years, Italy has received adults, young people, and children who, aboard boats, leave their countries of origin to reach Italian shores across the Mediterranean. The increase in the arrival of MENAS in Italy has led the government and international and civil society organizations to develop responses to improve reception, social inclusion, and the management of applications for international protection and residence permits. A significant development was the adoption of law number 47 of April 7, 2017, known as “Zampa law,” one of the most advanced measures to protect the rights of migrant and refugee minors. However, MENAS continues to face numerous difficulties in transit and upon arrival at their destination. According to the European Council Resolution of June 26, 1997, these are:

*“third-country nationals under 18 years of age who arrive on the territory of the Member States unaccompanied by an adult responsible for them by law or custom and until they are effectively taken into the custody of an adult responsible for them (…)”.*

**Figure 1:**
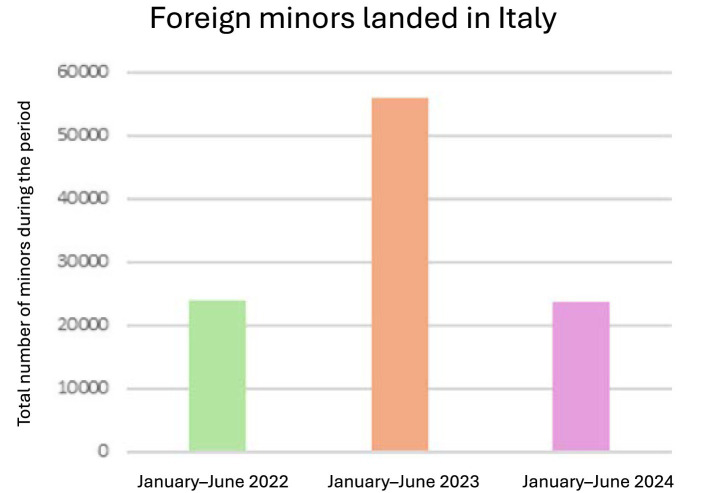
Foreign minors disembarked in Italy during the first semester of 2022–2024. Own elaboration.

The reorganization in favor of MENAS is also reflected in Italian laws, with Law 47/2017 and Legislative Decree number 220 of 2017, which seek to strengthen protection instruments. The Ministry of the Interior has released figures on the number of people who have landed on the coasts since the beginning of the year.^1^ To date, 23,725 unaccompanied foreign minors have landed on the coasts since the beginning of the year. In the same period, in 2023, there were 55,902, while in 2022, there were 23,920. On the other hand, the number of minors arriving on Italian shores by sea rose to 2,612 in June; in 2023, for the whole month, there were 15,164, while in 2022, there were 8,152 (Figure 1).

**Figure 2:**
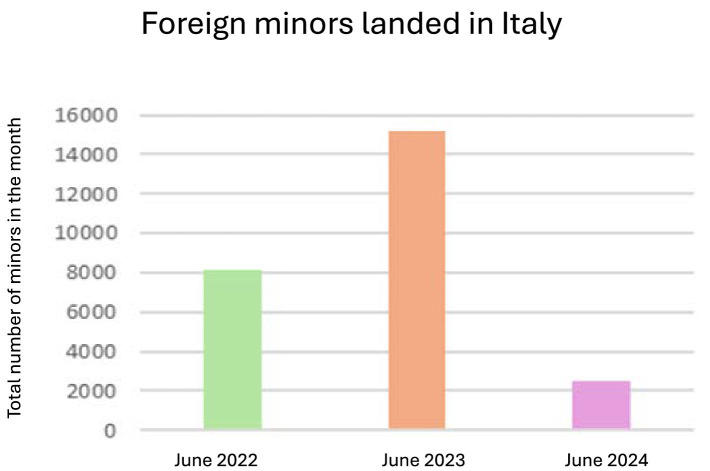
Foreign minors disembarked in Italy from 2022 to 2024. Own elaboration.

The statistical data from the research indicates that, while there has been a decrease in landings in 2024 compared to 2023, approximately half of the educational communities in Salerno are inhabited by unaccompanied foreign minors, with a proportion of over 13.4% of landings involving minors (Figure 2).

## 2 Elements for understanding the phenomenon of unaccompanied foreign minors

In accordance with the aforementioned legislative decrees, the authority to assign MENAS to families or communities rests with territorial juvenile courts. The reasons underpinning this phenomenon are of paramount importance. The immigration of minors in Italy is the result of multiple complex and interrelated causes, as highlighted by the United Nations High Commissioner for Refugees [United Nations High Commissioner for Refugees (UNHCR), 2020]. A significant proportion of these individuals are escaping conflict, war, and political, religious, or ethnic persecution in search of safety and protection. Poverty and economic hardship are also cited as key drivers of migration, with some children seeking better opportunities for themselves and their families (Save the Children, 2021). Furthermore, some adolescents migrate to access education or employment opportunities to hope for a better future [European Commission, 2018; International Organization for Migration (IOM), 2020]. However, it is equally important to acknowledge the tragic reality that some individuals are coerced into travel to Italy, only to be subjected to forced labor or prostitution against their will (U.S. Department of State, 2021). In conclusion, minors’ migration to Italy is a complex phenomenon driven by the need for safety, education, and better economic opportunities. However, this phenomenon also highlights challenges such as exploitation and abuse, underlining the importance of policies that guarantee their comprehensive protection. A reception center for minors, regulated in Italy by Law 184/1983 and revised by Law 149/2001, offers temporary shelter to minors who cannot stay with their families. These structures function through individualized interpersonal dynamics, facing continuous challenges to sustain internal and external relationships, as Cottrell (2013) points out. Bertani (2015) further elaborates on the multifaceted nature of these communities, highlighting their role in providing a secure environment for children while concurrently facing challenges arising from the expectations of various stakeholders. Children, in many cases, perceive these institutions negatively, seeing them as punitive. Parents hold divergent views on the value of these institutions as a source of support, with some considering them beneficial and others perceiving them as inappropriate, particularly when they feel that their parental competence is being undermined. Taurino and Bastianoni (2012) identify three categories of residential communities: immediate reception for situations of danger; family communities composed of couples and their children, possibly natural; and communities managed by teams of social workers who alternate in day-to-day management. Within these communities, a particular emphasis is placed on the provision of residence for unaccompanied foreign minors. This aims to offer these minors a serene environment to live in, express their potential, and find answers to their needs, providing them with opportunities they did not see in their home environment (Fondazione ISMU, 2019; ASGI, 2020). However, professionals involved in this process, including educators, psychologists, social workers, judges, and neuropsychiatrists, face various challenges related to relationships, bureaucracy, and education. Separating a child from their family environment is a complex but sometimes necessary measure to ensure healthy development, as stated in the Convention on the Rights of the Child (United Nations (UN), 1989).

Housing communities have been identified as environments that demand significant sacrifices from socio-educational actors. As Eckstein and Coll (1975) state:

*“The essence of communities is that, while each projects their inner world onto the macrosystem of the community, the operators find within themselves the strength to resist and not assume the transferential roles projected onto them” (p. 186).*

The placement of the minor, generally ordered by the Territorial Social Service under Article 403 of the Civil Code, is carried out on a provisional basis, formalized by the decree of the competent Juvenile Court. This mechanism is applied when the minor faces serious risks to their integrity or is in conditions of abandonment, both moral and material. Within 24 h of the intervention, the Territorial Social Service must inform the Minors’ Public Prosecutor of the reasons for the removal. Within 72 h, the prosecutor requests the Court to validate the decree, the deprivation of parental responsibility (Article 330 of the Civil Code), its revocation, or corrective measures for the parents (Article 333 of the Civil Code). The Juvenile Court validates the measure within 48 h, assigns a special guardian (usually a lawyer for the child), and sets a hearing within 15 days if the placement is confirmed. Family separation seeks to protect the child, ensuring that the child grows up in an environment conducive to positive and harmonious relationships.

Saglietti (2015) stresses that this intervention enshrines the child’s right to be raised in a family. If this is not feasible, temporary solutions such as family or community foster care are chosen, guaranteeing their education and basic needs. The authorities evaluate the family context considering the physical and emotional safety of the child, emotional balance, and social inclusion, prioritizing healthy relationships with adults and peers (UNICEF-UNHCRA-IMO, 2019).

### 2.1 Unaccompanied foreign minors: definitions and situation in Italy

Due to many disembarkations, the immediate reception structures, the Extraordinary Reception Centres (CAS), managed by the territorial Prefectures, have been reorganized. Medical checks are carried out there, with an initial stay of 60 days, extendable to 180 days. Subsequently, the minors are transferred to the Integrated Reception System (SAI), where they remain until 18, extendable to 21. In these structures, minors attend literacy courses in public schools to facilitate their integration. At the end of 2023, 293 unaccompanied foreign minors were registered in the Salerno region, many of whom are accommodated in host communities or CAS and SAI centers. A significant part of this group has relatives in the country, which could facilitate their inclusion in Italian society. However, the phenomenon remains a considerable challenge, as the majority are boys and represent more than 13% of all migrant minors in Italy. This underlines the need to strengthen reception and integration systems, ensuring these minors’ protection and proper development in a new socio-cultural context. The work of socio-educational agents with MENAS requires special and conscious attention to face the repeated experiences of loss and lack of acceptance, which mark these young lives, characterized by the discontinuity of living environments and the continuity of separation and derived anger (Bastianoni, 2000). According to the interactionist perspective, knowledge of oneself and the world begins at birth, with the I/Other differentiation, which develops in early interactions through self-referential (looking-glass-self) modalities, where the other becomes the mirror of existence (Cooley, 1901).

The educators’ approach can be described, according to traditional scientific parameters, as supporting the needs of users (Winnicott, 1965), “reverie” and the construction of thought in a context of action (Bion, 1965), catalyzing creative energies and identifying “horizons of meaning” (Fraire, 1970). In addition to the educational dimension, this work is characterized by the concepts of transference and countertransference. In this context, transference refers to the hidden desire that drives the child toward the educator, seeking “a satisfaction that is proposed again with each new object” (Novelletto, 1997). Conversely, countertransference is the inner resonance that the child’s transference provokes in the educator, being considered, when recognized, “the keystone of the educational relationship” (Novelletto, 1997).

Educational agents collaborating in educational communities play a key role in the social insertion of MENAS. However, they face several challenges when engaging with them. As Bion (1965) reminds us, they must rely on their ability to tolerate ambiguities and confusion, rejecting the search for tools that protect the socio-educational agent from the distressing experience of being alone in front of children. The operator should be prepared to face the complexity of the task, tolerating and understanding the feelings of bewilderment, helplessness, and frustration that arise daily. This dimension is made possible by the support of the working group, making it easier to cope with the situation’s complexity. Thanks to the encounter with the working group, particularly with the experiential group, the daily conflict situations can be overcome. If these conflicts are not recognized, they can become an obstacle to the professional relationship with the user. As Novelletto (1998) mention:

Given that the adolescent (…) has difficulties in distinguishing the internal reality from the external one, the operator (…) should be able to recognize, tolerate and process the passage from one side to the other, as long as it occurs in his/her relationship with the user (Novelletto, 1998, p. 69-70).

In short, it is only through teamwork that impasses can be overcome through mental operations that recover the sense of one’s value, expressing in each encounter the dynamics of daily professional collaboration, broadening self-awareness and relational and affective capacities (Montinari, 2001). According to Bastianoni (2000), problems of child maladjustment in housing communities can be addressed from a social perspective that values everyday relationships as supportive of development. Adults play a “scaffolding” educational role (Bruner, 1975; Sroufe and Cooper, 1988; Laicardi, 1995; Gilly, 1997) by facilitating tasks and interactions that children would not be able to cope with on their own, integrating themselves into everyday practices (Bickhard, 1992). Even with tutoring, scaffolding implies the adult’s regulatory action to reduce the child’s cognitive load (Bruner, 1985). Pontecorvo et al. (1998) define it as a particular form of mediation. The concept of “mentoring” emphasizes the psychosocial support and the guiding role of those more experienced in developing potential (Allen et al., 2004). Sharing meals in residential communities generates a protective environment that facilitates social learning, rebuilding opportunities for those who suffered traumatic experiences, and encourages coexistence, respect for rules, and the integration of diverse customs, contributing to the construction of a common society. The rules become an opportunity to learn social skills and promote respect for norms. The importance of the role of communities and socio-educational agents lies in the containment and listening to foreign minors before proposing activities. However, the fundamental task remains to provide rules for living and coexistence.

### 2.2 The “Nerverland” residential community for minors

Neverland was founded in 2016 as part of the Giovamente cooperative, a social cooperative established in Salerno in 2015 with the aim of promoting well-being and social integration through a wide range of services to meet the needs of the community. The activities carried out relate to support services for families, people with disabilities, unaccompanied foreign minors, and the care of minors. As a residential community, Neverland can accommodate up to eight minors between the ages of 13 and 18. To date, Neverland has offered unaccompanied foreign minors educational and psychological assistance to help them cope with the difficulties that every minor experiences, bringing out and expanding their personal resources. Through interaction with educational institutions, it initiates courses for learning the Italian language; it promotes social inclusion through workshops and sports activities; it provides career guidance to ensure effective integration.

Currently, the team consists of eight social workers, four women and four men (one with a degree in literature for academic support and Italian language learning; two with degrees in social work, one with a degree in psychology, two in political science, one specializing in cultural mediation, and one in social and educational services).

From 2016 to 2025, the Nervaland housing community has welcomed about 60 guests, all male, with an average age of about 16 and a half. The nationalities of the minors welcomed over the years are mainly Senegalese, Gambian, and Malian (from Central Africa), who arrived in Italy from the Libyan coast.

Since 2021, however, there has been a change in their origin: minors now arrive mainly from Bangladesh, Pakistan, Egypt, and Tunisia, following the Balkan route. The average stay in the community is about 1 year, especially in cases where the court authorizes continued accommodation until the age of 21.

The Neverland community has a process divided into stages. First, there is the pre-admission phase, where the child’s possible admission is assessed, the team studies the child’s file, and health screenings are carried out to protect the health of the child and other guests at the facility. Once this first phase is complete, there is the reception stage, during which the team draws up an Individualized Education Plan (IEP), through which they work with the minor to understand their needs and personal difficulties and to produce the necessary documents to ensure their successful integration into the host social fabric.

The third phase consists of literacy training. The minor is enrolled in public school to learn the Italian language and, at the same time, to create the conditions for obtaining the qualifications required for successful integration into Italian society. At the same time, the minor is directed to workshops that allow them to integrate into the social fabric and, once they reach the age of majority, to enter the workforce. The final stage, discharge, usually occurs when the objectives set out in the PEI are achieved.

## 3 Methodology

In the framework of a large project on residential educational communities, this study deals with the case of MENAS in Salerno, Italy. The reception center studied, managed by the cooperative “Giovamente” and known as Neverland, operates under laws 184/1983 and 149/2001. The aim is to reflect on the complexity of this social phenomenon, analyze the centers’ functioning and the agents’ actions in their daily work, and promote support and growth actions within the residential structures for minors. The qualitative research included in-depth interviews with the Salerno Juvenile Court judges, community agents, structure managers, Juvenile Prosecutor’s Office, law enforcement, and community residents, exploring their experiences and perspectives (Denzin and Lincoln, 2005). In total, 2 Juvenile Court judges, 1 Juvenile Prosecutor, and 2 representatives of law enforcement were interviewed, in addition to the 8 socio-educational workers involved in focus groups. The analysis considers the social, cultural, political, and economic influences surrounding this phenomenon (Yin, 2014). The research adopts an exploratory approach using case study and qualitative techniques to deepen the understanding of the specific context. Specifically, it focuses on the experience of different actors working in residential centers for these minors in Salerno. The research used various data collection techniques, such as focus groups, interviews, observations, documents, and cultural artifacts, to comprehensively understand the case (Creswell, 2013). In this study, the confidentiality of personal and contact details and the anonymity of participants were guaranteed, ensuring that the information obtained would be used exclusively for research purposes, in accordance with the provisions of the law on privacy for scientific research, regulated mainly by the Privacy Code (Legislative Decree 196/2003) and the General Data Protection Regulation (GDPR) of the European Union. To this end, informed consent was obtained prior to the interviews, in which participants stated that they had been informed about the study and the research procedures.

### 3.1 Procedure

Specifically, to respond to the research objectives, the methodology used is that of the case study (Yin, 2005), although aware that a generalization of the results is not produced, but nevertheless, it provides a datum that can become a focus of the characteristics of the object studied. In this research the case study was based on in-depth interviews done with a community educator, the coordinator of the center, each lasting about an hour, two focus groups to the entire team, consisting of 8 social education workers, 4 males with an average age of 45, and 4 females with an average age of 43. In the first focus gruop, semi-structured questions were asked, to bring out the needs of the socieducational agents and construct the categories reported in the research, the duration was about an hour; in the second focus group, however, the categories that emerged were explored in depth, the duration of this last meeting was approximately one and a half hours. For each meeting, there were moments of conviviality with the team within the Nerverland structure without the presence of the minors, during which, but, not only that, also during the focus groups and the entanglements, the researcher transcribed, at times, field notes. The construction of the empirical record took place from 25-03-2024 to 30-06-2024. The field notes (Cigliuti, 2014), were necessary to draw up an ethnological diary; the first, drawn up during and immediately after the meetings held with the socioeducational agents, had the intention of reporting the researcher’s impressions and exploring the experience of the socioducational agents, the experience in relation to the work and the educational approach used. The ethnographic diary constituted a fundamental reflective tool to record not only the observed facts, but also the researcher’s personal reactions, changes in subjective position. This made possible a deeper and more critical reading of the context, respecting the ethics of the research and the subjectivity of the participants. The research carried out consisted of several phases. The first phase included a focus group with eight community stakeholders to identify their roles and competencies. In addition, a mapping of the different social agents that collaborate daily in the communities was carried out, following the definitions of various authors (Acocella, 2008; Acocella and Cataldi, 2021). The second phase consisted of analyzing this map to explore the operators’ perceptions, the difficulties encountered, and the resources available in their interactions with different social actors, aiming to improve educational practices in residential interventions.

### 3.2 Categories of analysis

The analysis of the empirical material—comprising semi-structured interviews, focus groups, field notes, and an ethnographic diary—was carried out using a qualitative approach grounded in thematic analysis and the inductive construction of categories, following the principles of grounded theory (Charmaz, 2006; Glaser and Strauss, 1967). Coding was conducted without the use of computer-assisted qualitative data analysis software, due both to the epistemological orientation of the study and the nature of the dataset.

Specifically, it was determined that the volume of narrative material, while rich in meaning and complexity, was not so extensive as to require software-assisted analysis. Manual coding allowed for close engagement with the data and preserved the contextual and interpretive sensitivity needed for this type of inquiry.

This methodological choice is supported by three primary considerations.

First, it ensured a direct and reflexive relationship between the researcher and the data, fostering awareness of nuances, emotional tones, and latent meanings. Second, the manual and iterative annotation process allowed the researcher to maintain full control over the interpretive logic, avoiding fragmentation or over-reliance on mechanized segmentation. Third, this form of analysis served as a formative and situated learning process, aligning with the ethnographic posture of the research and enabling deeper involvement with the lived experiences narrated by participants.

The construction of categories followed three successive phases:

Open coding involved the initial segmentation of the transcripts into meaningful units, assigning descriptive labels to salient passages without relying on predefined analytical grids. This phase privileged participants’ voices and encouraged spontaneous emergence of thematic patterns.

Axial coding reorganized the initial codes into broader conceptual categories by identifying relationships among recurring themes, taking into account their frequency, contextual relevance, and resonance across different data sources (interviews, field notes, group discussions).

Selective coding led to the identification of six main thematic categories, selected for their explanatory power and recurrence within the dataset:

(1) the map of social actors;
(2) motivations for migration;
(3) challenges in community-based educational work;
(4) the role and function of the socio-educational practitioner;
(5) the perspective of unaccompanied minors;
(6) operational tools.

These categories did not result from *a priori* assumptions but emerged from an iterative and reflective reading of the data, employing the constant comparative method and ensuring internal coherence through triangulation across sources. The ethnographic diary served as a vital tool for documenting the researcher’s interpretive choices, doubts, emotional responses, and evolving understanding throughout the analytic process (Cigliuti, 2014).

The resulting categories thus operate not merely as thematic groupings, but as interpretive devices that reflect the emotional, relational, and institutional complexities of educational work with unaccompanied minors. They offer a situated and nuanced understanding of professional practice within residential care settings, highlighting the need for shared meaning-making and reflective postures among socio-educational teams.

## 4 Analysis of results

The structural elements of a reception center are reflected in the dynamics of internal relationships, a space to experience personalized bonds, appropriate the environment, and maintain external ties. It is conceived as an opportunity for children, a transitional period that enhances past relationships with present one’s previous knowledge with new ones and facilitates the positive restructuring of everyday life. Understanding a reception center involves analyzing its members. As indicated by socio-educational agent 1:

*In the center, there are the children, the users that we accommodate, and around them are the operators of the structure, the family of origin, the person in charge of the structure, the cultural mediators, the social worker of the structure, the psychologist or the counselor, the social worker of the municipality.*

The social role of the center is conditioned by the needs of the children and their relationship with social actors present in the territory, as highlighted by socio-educational agent 2:

“*Such as the family, the school institution, the social services, the Court, the Police Station, the Embassy, the Local Health Company, the Neighbourhood, and the recreational centers.”*

The socio-educational agent 6 adds:

*“For me, an educational community is made up of continuous challenges. The most critical challenge is to be accepted by young people and the territory. The more difficult the relations with the territory are, the more the challenge of integration increases; therefore, for me, overcoming the challenges with the territory and with the children makes community*”.

From the field notes “*all relationships with institutions are opportunities for networking and opportunities for educational practice even if bureaucracy and communication are often not always effective and do not make the work easie”r*.

Socio-educational Agent 5 states: “*through the personal network I was able to get to the teachers and thus facilitate the placement of minors in school. The part that works the least is the fact that there is not always communication between the* var*ious social agencies”*.

The center represents a key resource within a complementary network of services, offering appropriate and targeted responses through interaction with agencies in the territory. Despite obstacles, its capacity to integrate external entities makes it a valuable social resource. The community must be an environment where daily life and activities are integrated, recreating a “socio-relational” climate like that of the family, emotionally and materially supporting the growth of each child, especially those foreigners without family references. The community for minors exists to respond to social and educational needs. Compared to other services, its role is to guarantee accessibility to resources, promote participation in meaningful projects, and facilitate cultural integration. Socio-educational agents, aware of their role, must create a relational environment, promote cultural integration, and offer opportunities in the territory, ensuring a dignified daily life. The shelters for MENAS form a complex network of actors, including agents, families of origin, caregivers, cultural mediators, and minors. These spaces provide a safe and nurturing environment, promoting young people’s personal and social development. With their dedication and skills, the socio-educational agents face challenges and maximize opportunities, ensuring each child receives the support needed to thrive. The migration of MENAS to Italy is a complex and multifaceted phenomenon. The reasons that lead them to leave their countries have changed over time, as the Coordinator of the Educational Community explains:

*The reasons that lead children to leave their country are diverse. Initially, minors arrived on Italian shores because of wars and poverty. Over time, things have changed; in fact, foreign children now come to Italy because of climate change. Children from Pakistan and Bangladesh speak of poverty related to climate change, which has dramatically affected their economy and, therefore, their livelihoods. Those from families of artisans or farmers can no longer continue the work their parents or grandparents did precisely because of climate change. In addition, globalization and, in particular, the use of the internet has created in young people the idea of being able to change their way of life, favoring migration.*

Some young people migrate to avoid specific situations, such as compulsory military service, according to socio-educational agent 2:

*Many young people have decided to embark on a long, dangerous, and sometimes traumatic journey and land in our country. It has happened several times that Egyptian minors arrive in Italy to avoid compulsory military service. In fact, it is rare for a fifteen-year-old Egyptian minor to arrive in Italy; it is usually the seventeen-year-old, close to the age of majority, who arrives on our shores and then leaves when the obligation has ceased in their country of origin. These minors pay large sums of money to reach Italy.*

Other motives include the desire to change their situation, as mentioned by socio-educational agent 4:

*“Many minors come to Italy because they want to change their situation, they make huge sacrifices, they risk, lives, they leave their families. Often these kids tell absurd, unspeakable things”.*

During focus groups and interviews, social workers consider the migration of minors mainly due to economic factors, environmental, and social factors. Climate change, poverty, globalization, and avoiding obligations such as military service drive these young people to seek a better future in Italy. The stories shared by the professionals highlight the sacrifices and risks taken by the minors, underlining the importance of a deeper understanding and support to face the challenges of this migratory reality. Working with MENAS presents multiple challenges for socio-educational agents, stemming both from the traumatic experiences of minors and organizational and contextual difficulties. These complications are reflected in the comments of the professionals interviewed.

Socio-educational Agent 3 states:

*“My greatest difficulty is not always achieving the objectives set for each child,” while Socio-educational Agent 7 underlines: “The biggest difficulties I have are when the child does not cooperate, due to their lack of motivation. Some children don’t want to follow any path, sometimes out of apathy, and I feel powerless”.*

From the field notes: “*Minors do not always follow the rules and this becomes a problem. Sometimes, due to their experience they already feel great, they have still gone through difficult situations to get to Italy, therefore, despite their young age, they feel independent and have difficulty complying with the rules of a community*”.

The Education Community Coordinator highlights another fundamental aspect:

*A significant difficulty that an operator might have is related to the expectations that one has on the child, and therefore, the educator must free themselves from that expectation. Sometimes, it is a project that the operator chooses for the child. Whatever the operator chooses whatever project he has in mind must be shared with the child and the whole team; only in this case will there be benefits and growth for the child. Otherwise, one loses sight of the goal to which one is called.*

The housing community operator must free himself from a series of interpretations, judgements and prejudices and listen to the person in front of him. Often, an operator’s judgments and prejudices can be linked to the country of origin, i.e., the beliefs acquired about a given country and allowing oneself to be conditioned. This behavior jeopardizes the operator’s performance, putting them in unfavorable situations.

Thus, these challenges can be both practical and emotional and require specific skills and adequate resources to be managed effectively. Difficulties also include communication barriers, compounded by linguistic and cultural differences, and situations related to trauma and psychological stress for children. Many MENA have suffered traumatic experiences such as conflict, abuse, or dangerous journeys, which can lead to disorders such as anxiety, depression, or post-traumatic stress disorder (Attanasio, 2018; Simeone, 2022). According to Priebe et al. (2016), psychosomatic disorders, anxiety, psychosis, depression, and substance use are common. In addition, the main challenges faced by a socio-educational agent have to do with the perception of judgments and prejudices linked to stereotypes, but above all, the knowledge of limits must be considered to maintain the appropriate distance and improve the interaction with the children present in the structure and with the work team, as Banks (2012) affirms. Socio-educational actors must be prepared to manage these situations, which require specific training and adequate resources. Several studies indicate that MENAS are particularly vulnerable due to various individual factors and ongoing exposure to violence and traumatic events before and during migration. These events are often associated with symptoms of psychological distress (Bean et al., 2003). In addition, separation from family members and support networks is another risk factor (Hebebrand et al., 2016). Other challenges mentioned by agents include bureaucratic management: “The first time I had to get documents at the embassy for a minor was a tragedy. I could not find anyone who could give me information or tell me what to do” (Socio-educational Agent 1). In addition, a lack of clarity on the age of minors can complicate access to rights and services, as mentioned by Socio-Educational Agent 8:

*Many minors do not have the age they declare, and sometimes they do not know it sometimes they hide it because if they were of legal age, they would not benefit from some rights reserved to minors, so they have difficulties following the rules imposed on minors under a certain age. Unfortunately, there is no way to find out the real age; even the medical examination of the wrist x-ray to orientate and give a chronological age is unreliable, as it recalls an approximate age but not a precise one.*

Furthermore, actors must facilitate the social integration of MENAS into Italian society, which represents a significant challenge. This includes school insertion, participation in social and cultural activities, and building support networks. Socio-educational actors need to overcome prejudices and foster an inclusive environment. Processing documents for legal residence, asylum, or other forms of international protection is often complex and time-consuming. They must deal with a usually cumbersome bureaucratic system that guarantees children’s rights. Communities and organizations working with MENAS operate with limited resources regarding staff and funding, reducing the capacity to provide adequate and quality services. The various actors need emotional support to prevent burnout. Working with vulnerable children can be emotionally draining due to constant exposure to stories of suffering and the difficulty of making a difference in challenging contexts. Operators must have access to psychological support and recovery moments. The effectiveness of actors’ work also depends on collaboration with institutions such as schools, health services, local authorities, and NGOs. Coordinating these efforts to ensure common goals can be a significant challenge.

### 4.1 Weaknesses of socio-educational actors

Socio-educational actors face challenges and have weaknesses, as indicated by some interviewees. Socio-Educational Agent 3 mentions that one of these is “not to get involved in children’s stories, but to dose appropriate participation and fruitful distance, to avoid burnout.” Socio-Educational Agent 6 points out that “working too much in a group can become a limit for the socio-educational agents working. It can make us resemble the thinking of others, losing sight of our own individuality.” Furthermore, Socio-Educational Agent 8 underlines the importance that “an operator must always be present to himself, maintaining a balance between feeling too close or too far away, both from the working group and from the group of minors present in the structure.” These comments reflect the need to address these difficulties through ongoing training, adequate support, and policies that recognize the work of social workers with MENAS and promote a multidisciplinary approach to meet their needs comprehensively. The socio-educational agents’ perceptions of the essential functions of their role highlight the importance of relational and communicative aspects in their work with children. Several interviewees expressed the following.

Socio-Educational Agent 2: *“The most important function that a socio-educational operator must have is empathy.”*

Socio-educational Agent 5: *“For me it is the ability to communicate also and above all with listening.”*

Socio-educational Agent 4: *“Honest and clear confrontation with minors. Communicating through reciprocal active listening.”*

Socio-educational agent 7: “For me, it is the example that every educator puts into practice through the daily shift in the community. The children follow the example that everyone sets.”

These fundamental functions reflect the fact that the educator must be a role model, as Bandura (1977) points out, and that active listening is crucial for effective interaction with children.

Unaccompanied minors’ perceptions of socio-educational actors in residential communities vary depending on various factors such as their personal experience, support received, and cultural differences. Many children who have been through traumatic experiences perceive the operators as key figures in their adaptation. As one resident of a community for unaccompanied minors who has reached the age of majority recounts (M01):

*In 2017, I left my country. I passed through and Sudan, and then I arrived in Libya. I stayed in Libya for about a year, and then I reached Italy by boat. It was a very difficult journey — it lasted three days. We landed in Sicily in 2020. At first, I stayed in a reception centre in Sicily, then I was transferred to a community in the province of Salerno, and finally to this community. Here, I felt good. The staff and the socio-educational workers are very kind — like a family to me. My family stayed in my country, and I’m alone in Italy, alone here in Salerno (…). The community workers are like family. They helped me with my documents, supported me in many things, and were a great example — let’s put it that way. They helped me go to school and learn Italian. Thanks to them, I found a job — I’m working as a waiter. I’m very grateful to the staff in this community for everything. Now I’m leaving the community. I’ve already rented a room in a shared house, and I will be living with other guys, each of us in our own room.*

The experience of mirroring is crucial for the development of the self and the integration of unaccompanied minors, especially when they have gone through traumatic situations such as long and arduous journeys. The lack of this process can be profoundly damaging, affecting their ability to adapt to a new environment with an unfamiliar language and culture. Initially, the child may accept removal from their family to escape painful and dangerous situations, but over time, the need to find substitute figures who represent security and trust becomes fundamental to their emotional development. Many children regard socio-educational actors as key support figures, providing them with security and guidance in times of vulnerability. They often thank for the emotional and material support received, recognizing its importance in adapting to a new life. As the interviewee mentions:

*“I have often felt and still miss my parents, although the socio-educational operators made me feel welcome, directing me in everything”.*

Children who develop positive relationships with socio-educational actors often feel trust and affection, seeing them as reference figures and role models. These bonds contribute to their sense of security and well-being. However, some children may perceive workers as authority figures, like parents or teachers, which generates respect but can also lead to resistance or conflict if they feel their autonomy is limited. Sometimes, frustrations or misunderstandings arise due to language or cultural barriers and rigid rules and routines, which may be perceived as restrictive and lead to dissatisfaction or conflict. In some situations, children may face negative experiences, such as feeling discriminated against or mistreated, if they do not receive the respect and understanding they need. Therefore, communities must constantly monitor children’s well-being and address their grievances. An inclusive and welcoming environment, supported by professionals committed to building trust, encourages the development of a strong sense of belonging. Recreational, educational, and cultural activities can facilitate integration, although some children, especially older ones, may desire greater autonomy, leading to tensions with community rules. In summary, the relationship between children and socio-educational actors is complex and multifaceted and is influenced by the support offered, the empathy and professionalism of the actors, and the expectations of the children. Listening to their needs is essential to foster an environment of mutual respect. For the socio-educational agents working with MENAS, having the right tools and approaches is key to their work. Socio-educational Agent 2 stresses that: “empathy, prior information about the minor, knowledge of the minor’s culture of belonging, resources, and networks present in the territory,” while Socio-educational Agent 5 considers that “it can be listening to the minor and his or her needs.” Another interviewee, Socio-Educational Agent 8, mentions: “For me it is what is done with the children. Each operator will have to adapt to the needs of the child, respecting the relationship that has been established, and the time spent getting to know the child is also very important.” According to Socio-Educational Agent 3, useful tools refer to: “the ability to communicate, to manage conflicts effectively, to use physical and psychological energies well to engage in work.” On the other hand, the following are mentioned as applicable: “the user’s motivation to change, the professionalism of the operator” (Socio-educational agent 6), as well as “the confrontation with the group of operators, the socio-educational agents, the psychologist and the person in charge of the structure” (Socio-educational agent 1).

Another interviewee, Socio-Educational Agent 4, underlines:

*For me the useful tools for children to experience their knowledge and acquire relational skills are: mealtimes, taking turns cleaning the house, washing dishes, homework, getting to know their friends at school or the knowledge acquired during the activities they do.*

The comments reflect key approaches and tools for socio-educational actors in their work, highlighting empathy, active listening, adaptation to individual needs, effective communication, professional support, and integration of daily activities. These elements are fundamental for the well-being of children. According to Neri (2019), the most essential tools are empathy, knowledge of the child’s culture, and time-sharing, which are crucial to achieving the established objectives. In the communities, socio-educational agents follow an explicit code of values that allows children to choose a guide that makes them feel welcome without a rigid imposition. They prefer an adult who listens to them and adapts to their needs. Operators play a fundamental role in establishing flexible and effective relationships, promoting an intervention that integrates the youngest with the youngest, and favoring an atmosphere of trust and support. Taking an active part in daily life, organizing and personalizing living spaces helps to take control of these and of the emotional aspects, often already put to the test. Workers in shelters for children with disabilities use a variety of tools to carry out their work effectively. Access to policy documents on the treatment of children, including national and international laws, safety protocols, and standard operating procedures, is essential. Socio-educational actors need continuous training to understand children’s needs and improve their skills with vulnerable children. Tools such as questionnaires, structured interviews, and psychological testing are required to assess children’s needs upon arrival and during their stay in the community. In addition, resources should be available to provide psychological and social support, such as counseling, emotional support, and educational resources. Establishing links with other organizations and professionals, such as social, legal, educational and health services, is also key. Technological tools can help manage documentation, communicate with other professionals, and monitor children’s welfare. This can include case management software, online communication systems, and mobile devices for quick access to information. Operators working with unaccompanied foreign minors (MENAs) should demonstrate sensitivity to cultural and linguistic differences and possess skills in intercultural communication and interpretation. It is essential to ensure that minors can access educational and recreational opportunities, including learning materials, leisure activities, sports, and cultural programs. While the implementation of such instruments may vary depending on the context and available resources, they represent a valuable set of tools for socio-educational professionals operating within host communities. MENAs face a range of challenges, including social integration, access to education, and the need for psychological and legal support. Educational interventions, such as intensive language courses, are particularly beneficial within housing communities as they facilitate integration. Additional educational support may include tutoring in core academic subjects, assistance navigating the school system, and vocational training to enhance future employment opportunities. Psychological support is also of paramount importance. This includes access to trauma-informed care pathways, group therapy sessions, and peer support initiatives designed to foster emotional well-being. Legal assistance should encompass providing clear and accessible information on asylum procedures and citizenship rights and referrals to qualified legal professionals. Promoting the social integration of MENAs can be further supported through participation in cultural and recreational activities and mentoring programs involving local volunteers who can provide guidance, emotional support, and relational continuity. For those approaching independent living, financial literacy programs can offer practical skills for autonomy. Moreover, community awareness initiatives and partnerships with local authorities and non-governmental organizations are crucial for strengthening the broader support network. Finally, ensuring access to digital tools—such as internet connectivity and computers—is essential to enable minors to pursue educational opportunities, maintain contact with family members, and develop digital competencies.

Implementing these measures requires a coordinated, multidisciplinary effort involving educators, psychologists, legal professionals, social workers, and volunteers working collaboratively to create a safe, supportive, and inclusive environment for unaccompanied minors.

## 5 Discussion and conclusion

The analysis conducted, in the Nerverland community, highlights how the work of socioeducational agents within the housing communities for minors is profoundly marked by a relational, emotional and institutional complexity.

The theoretical framework based on social learning (Bandura, 1977), transference and countertransference dynamics (Novelletto, 1997), and the notion of scaffolding (Bruner, 1975) proved particularly useful in interpreting the empirical findings. For instance, the emphasis placed by socio-educational agents on empathy, active listening, and role modeling directly reflects Bandura’s perspective on learning through observation. Similarly, the recurring tension between emotional closeness and professional distance can be read through the lens of transference and countertransference, highlighting the relational and affective complexity of daily practice. Finally, the centrality of teamwork and shared activities such as meals resonates with Bruner’s concept of scaffolding, where structured support fosters autonomy and integration. These theoretical insights thus provide a coherent interpretive lens for understanding the practices and challenges emerging from the fieldwork. Moreover, the empirical findings demonstrate how these theoretical models converge in practice: socio-educational agents embody Bandura’s model of learning through role modeling; the structured support provided through shared activities and teamwork reflects Bruner’s notion of scaffolding; and the emotional balancing between empathy and professional detachment exemplifies Novelletto’s framework of transference and countertransference. This interplay reveals that theoretical and practical dimensions are mutually reinforcing, allowing the agents to transform pedagogical theories into daily, relational strategies within the community setting. Analyzing the categories that emerged, and precisely from the extracts, we denote a circular representation centered on the minor, which highlights the structure of the Neverland reception system as a complex and interdependent network or map of figures. Children and/or young people are not isolated subjects, but are considered to be at the center of a constellation of professional, institutional and family relationships. This suggests a systemic perspective in socioeducational work. The importance of coordination between actors, for Nervertal, guarantees the protection and development of the minor, as well as his insertion into the social fabric. This integrated vision, for Nevertal, takes into account the various levels (micro, meso, macro) of the educational context. Through this map, the institutional actors involved detect a weakness of communication between the various institutions, which often becomes a limit for the work of socioeducational agents.

Moreover, in analyzing the educational difficulties, drawn from the excerpts, it can be observed how the emotions aroused by the educational relationship –often ambivalent and difficult to process – are situated at the intersection of individual psychological dimensions and collective cultural constructions, helping to define the quality of the socioeducational intervention. Among the educational difficulties, which emerged from the extracts, of the socioeducational agents of the Nevertal Community, there are “the psycho-emotional fragilities” of minors, which must be managed by a strong balance between empathy and professional distance (Milani, 2006), as declared in the category of the educational function. It is useful to note, from the perspective of cultural psychology and the anthropology of education (Shweder, 1991; Spittler and Bourdillon, 2012), how often the relationship and interaction between community guests and socioeducational agents, can reflect not only traumatic experiences and the search for substitute figures of attachment, as it emerges from the interview with the young community guest who has come of age, but also from the influence of cultural narratives related to authority, to care and belonging. Given the interaction in these terms, we could speak of the transference of minors in search of figures to replace and the countertransference of socioeducational agents. While the former project their shortcomings, countertransference in socioeducational agents can be considered as an expression of intercultural empathy and processes of identity “counter-narration” (Delgado, 2003; Dei, 2006).

The weaknesses, but also the strength of the educational work, lie, for the agents of Nervetal, on the group boundary of the team, in fact, from the extracts, some important points of the group work activity emerge, the team can be a resource, but also a limit for the work of the socio-educational agents. If on the one hand the working group helps to find new strategies, to share the sense among educators and to find the systematic comparison on daily practices, contributing to the construction of a reflective posture, capable of sustaining the complexity and educational responsibility (Mortari, 2003), sometimes it becomes limiting for the action of the individual worker. Just as the emotional load implicit in educational work often generates experiences of loneliness and frustration, fueled by a poor culture of supervision and fragmented shifts (Riva, 2011). Added to this is a certain marginality and a weak recognition of one’s professional mandate (Kaneklin & Scaratti, 2012).

It is interesting to note how the categories “useful tools” to the operator coincide with “the function” that they have. “Empathy, communication, example to follow” are characteristics that at the same time are a function and tool of socio-educational agents. From the extracts it is clear that “doing” and “tools” are often used automatically. It lacks, therefore, what Gouldner (1997) defines as: “the seen but not recognized”; a reflection on action within action itself, what Schoen described as reflection on one’s own practice, as a mode of learning.

Aware of all the limitations of a case study, the small number of participants and the weakness and limitations that the research presents, it is believed, however, that the present investigation can be a tool for reflection on the work of socioeducational agents who work within residential structures, also because to enter a community for minors the researcher needs contacts, time and resources, since the level of protection that binds these contexts is very high.

It is no coincidence, in fact, that many of the research that has been conducted in communities for minors are carried out by educators who work there, or by people who are in different capacities involved in associations that manage communities for minors (Bastianoni and Palareti, 2005; Zullo et al., 2008; Bastianoni and Taurino, 2009). Furthermore, doing research in a context where there is continuous change (think of minors who have come of age and who have to leave the community or of minors who flee from it, or of the simple shift of socio-educational agents) becomes tiring and grasping its facets it is always difficult. This is why data must be collected very quickly, as situations change rapidly. If qualitative research has many advantages, there are not so few weaknesses, just think of the analysis of the data and the time it takes to encode them. On the other hand, however, this work and with the various techniques allows the construction of the social context, inevitably partial, but equally precious.

In conclusion, the educational work, in a community for unaccompanied minors, consists of the possibility, that the socioeducational agents can educate themselves while educating, in a process that takes the form of affectionate welcome, mutual trust, respectful and attentive listening, for themselves, the team to which they belong, the unaccompanied foreign minors. Only in this way is it possible to guarantee truly transformative interventions, capable of offering welcomed minors not only protection, but also significant evolutionary opportunities.

## Data Availability

The original contributions presented in the study are included in the article/supplementary material, further inquiries can be directed to the corresponding author.

## Footnotes

1
www.interno.gov.it
